# White Adipose Tissue Response of Obese Mice to Ambient Oxygen Restriction at Thermoneutrality: Response Markers Identified, but no WAT Inflammation

**DOI:** 10.3390/genes10050359

**Published:** 2019-05-10

**Authors:** Femke P. M. Hoevenaars, Jaap Keijer, Inge van der Stelt, Loes P. M. Duivenvoorde, Laure Herreman, Robin van Nes, David Friedecký, Maria A. Hegeman, Evert M. van Schothorst

**Affiliations:** 1 Human and Animal Physiology, Wageningen University, P.O. Box 338, Wageningen 6700 AH, The Netherlands; femke.hoevenaars@TNO.nl (F.P.M.H.); jaap.keijer@wur.nl (J.K.); inge.vanderstelt@wur.nl (I.v.d.S.); loes.duivenvoorde@wur.nl (L.P.M.D.); laure.herreman@hotmail.fr (L.H.); robinvannes@hotmail.com (R.v.N.); m.a.hegeman@uu.nl (M.A.H.); 2 Laboratory of Metabolomics, Institute of Molecular and Translational Medicine, University Hospital Olomouc and Faculty of Medicine and Dentistry, Palacký University Olomouc, Hněvotinská 5, 779 00 Olomouc, Czech Republic; david@friedecky.cz

**Keywords:** hypoxia, whole genome microarray gene expression, cholecystokinin, white adipose tissue, inflammation, adipokine

## Abstract

Obesity is associated with white adipose tissue (WAT) hypoxia and inflammation. We aimed to test whether mild environmental oxygen restriction (OxR, 13% O_2_), imposing tissue hypoxia, triggers WAT inflammation in obese mice. Thirteen weeks diet-induced obese male adult C57BL/6JOlaHsd mice housed at thermoneutrality were exposed for five days to OxR versus normoxia. WAT and blood were isolated and used for analysis of metabolites and adipokines, WAT histology and macrophage staining, and WAT transcriptomics. OxR increased circulating levels of haemoglobin and haematocrit as well as hypoxia responsive transcripts in WAT and decreased blood glucose, indicating systemic and tissue hypoxia. WAT aconitase activity was inhibited. Macrophage infiltration as marker for WAT inflammation tended to be decreased, which was supported by down regulation of inflammatory genes *S100a8*, *Ccl8*, *Clec9a*, *Saa3, Mgst2,* and *Saa1*. Other down regulated processes include cytoskeleton remodelling and metabolism, while response to hypoxia appeared most prominently up regulated. The adipokines coiled-coil domain containing 3 (CCDC3) and adiponectin, as well as the putative WAT hormone cholecystokinin (CCK), were reduced by OxR on transcript (*Cck*, *Ccdc3*) and/or serum protein level (adiponectin, CCDC3). Conclusively, our data demonstrate that also in obese mice OxR does not trigger WAT inflammation. However, OxR does evoke a metabolic response in WAT, with CCDC3 and adiponectin as potential markers for systemic or WAT hypoxia.

## 1. Introduction

Obesity is the result of long-term excess energy intake over expenditure, resulting in an increase in white adipose tissue (WAT) mass to store surplus energy as triacylglycerides. This essential lipid storage function of WAT protects the body against lipotoxicity [1]. To perform its role as a dynamic storage depot, WAT produces and secretes a wide range of adipokines, including leptin and adiponectin, that are involved in homeostatic regulation [2,3,4]. However, it was also shown that the pro-inflammatory adipocytokine TNF-α can be synthesized and secreted by WAT [5]. This provided the first direct link between WAT and low-grade inflammation. WAT inflammation is one of the signs of impairment of WAT function. Morphologically, WAT inflammation is visible as an increase in the number of so-called crown-like structures (CLS), macrophages that are localized around dead adipocytes [6]. WAT inflammation is thought to contribute to obesity-associated metabolic complications like insulin resistance and hyperinsulinemia, leading to type 2 diabetes, cardiovascular diseases, and the metabolic syndrome [7,8]. As these diseases have a large societal and economic impact, it is important to know the triggers that contribute to the change from a healthy WAT to a dysfunctional, inflamed WAT. Of note, a subset of obese individuals is metabolically healthy, and remains insulin sensitive [9,10]. This indicates that not only the amount of WAT, but also other triggers initiate the inflammatory state of WAT. One such trigger may be an inadequate oxygen supply (hypoxia), as has been postulated [11].

Obesity is associated with reduced body oxygen supply to peripheral tissues as a result of increased body volume and a decreased breathing capacity of the lung [12]. WAT oxygen supply in obese individuals is further limited by a lower WAT capillary density and a lower WAT blood flow [13,14], compared to normal weight individuals. Furthermore, an increased adipocyte size is thought to contribute to WAT hypoxia [15]. Indeed, several mouse and human studies demonstrated that hypoxia occurs in obese WAT [16,17,18,19]. Co-localization between hypoxic areas and macrophage infiltration suggests a direct link between hypoxia and WAT inflammation [17]. This was supported by the association in obese subjects of a lower WAT blood flow and impaired vascularization with increased insulin resistance and a higher gene expression of inflammatory markers [20]. Unexpectedly, using micro-dialysis this study observed a higher oxygen partial pressure in WAT, which was likely due to a lower WAT oxygen consumption and a decrease in gene expression of mitochondrial markers that was observed in obese compared to lean individuals [20], while others reported a decrease in obese WAT oxygen pressure by hypoxic exposure concomitant with improved glucose homeostasis [21]. Nevertheless, a decreased WAT mitochondrial density is widely observed in obese WAT [22]. Thus, in obesity, impaired WAT displays signs of tissue hypoxia as well as decreased oxygen consumption, reduced mitochondrial density, and signs of inflammation. While these phenomena are observed, cause and effect are less well established; it is, for example, not known whether hypoxia is a cause or consequence of WAT inflammation in obese conditions.

Therefore, we decided to examine in this study whether hypoxia triggers WAT inflammation in obese conditions or whether also then inflammation is repressed. For this, diet-induced obese (DIO) male mice were exposed to environmental oxygen restriction (OxR) of 13% O_2_ for five days. We assessed body composition, systemic metabolic parameters and adipokines, epididymal WAT inflammation, and performed whole genome transcriptomics of WAT to examine effects induced by mild environmental hypoxia. Our results showed that also in obese mice, environmental hypoxia does not increase the inflammatory state of WAT, while it evokes a WAT metabolic response.

## 2. Materials and Methods 

### 2.1. Animal Study

Male wildtype C57BL/6JOlaHsd mice, aged 9 weeks, were purchased from Harlan (Horst, The Netherlands). Mice were acclimatized for three weeks to thermoneutrality (29–30 °C, 12 h light/dark cycle, 55 ± 15% humidity). During these three weeks mice were fed a semi-purified low fat diet, followed for 13 weeks by a semi-purified high fat diet (Research Diet Services, Wijk bij Duurstede, The Netherlands) according to [23], detailed composition in Supplementary Table S1). Mice were housed at thermoneutrality to increase adipocyte size and to exclude thermogenic metabolism as a confounder when challenged by hypoxia [24]. Access to water and food was ad libitum and renewed every week. Body weight, food consumption, and body composition (EchoMRI 100V, EchoMedical Systems, Houston, TX, USA) were measured weekly from week 7 onwards. After 11 weeks of high fat feeding, the DIO mice were stratified on fat mass into a control group (C, n = 12), an OxR group (n = 18), and a group which was not part of the current study [25]; data of body weight, lean and fat mass, and blood glucose levels of the control group were reported previously [25]. At the end of week 12, OxR mice were acclimatized to indirect calorimetric chambers for 48h under normal ambient 20.9% oxygen, followed by exposure to 13% O_2_ as described [26], with the following adaptations: mice remained under hypoxic conditions for 120 h with ad libitum access to food and water. We choose to use 13% O_2_, since this is a level of oxygen that individuals may encounter when staying at high altitudes (~4000 m), flying in an airplane, [27] or as a result of apnoea [28]. Mice were killed directly thereafter to collect tissues under hypoxic conditions. Six mice were used pre- and immediately post-hypoxic exposure only for body composition analysis and not taken along for tissue and serum analyses. Activity levels were measured by infrared light-beam frames surrounding the cages. Principles of laboratory animal care (NIH publication no. 85-23, revised 1985) were followed, and all animal care and use was according to the guidelines given by the Dutch experimentation act (1996). Permission for this study was granted by the Animal Ethical Committee of Wageningen University (DEC2012056).

### 2.2. Tissue and Blood Collection

Food was removed 2 h before the mice were killed in the morning (light phase). Mice were directly killed by decapitation, and blood was partially collected in 60 µL heparinised capillary tube (Hirschmann Laborgeräte, Eberstadt, Germany) for determination of whole blood haematocrit levels. Capillary tubes were centrifuged in a micro-haematocrit centrifuge at 3000× *g* for 5 min. Haemoglobin (Hb) levels were measured using an automated Hb monitoring system (Hemocue 201 Plus, HemoCue Ltd., Angelholm, Sweden) with 10 µL micro cuvettes. Remaining blood was collected via a funnel into mini collect serum tubes (Greiner Bio-one, Longwood, FL, USA) and spun down 10 min at 5780× *g* at 4 °C. Epididymal WAT (eWAT) was excised rapidly, left portion weighed and snap frozen in liquid nitrogen. The right eWAT portion was fixed overnight in 4% paraformaldehyde, washed with PBS and paraffin embedded for histological analysis. We focused on eWAT because it is the most widely examined WAT depot in mice, it is a visceral AT depot and visceral AT is most strongly associated with disease risk, and eWAT responds first to an obesogenic condition.

### 2.3. Tissue Analysis and Histology

Lactate in eWAT was determined using the Lactate Assay Kit II (Biovision, Mountain View, CA, USA) according to the manufacturer’s protocol. Mitochondrial density markers citrate synthase and aconitase activity were determined as published [24]. Paraffin embedded eWAT was sliced at 5 µm and stained with a MAC-2 antibody recognizing macrophages for detection of CLS as published [29], and using MAC-2 fluorescence staining for individual macrophage counting. HE-stained WAT sections were used for determination of adipocyte size distribution as published [24].

### 2.4. Serum and Tissue Adipokine/Insulin Analysis

All serum protein measurements were done using Bio-Rad materials and associated protocols (Bio-Rad laboratories, Veenendaal, the Netherlands), unless stated otherwise. For adiponectin, the Bioplex Pro mouse adiponectin kit was used, which detects total adiponectin.

For determination of tissue adiponectin, total protein was extracted from 50–100 mg WAT with 2 µL cell lysis buffer per mg tissue. After a freeze-thaw step, homogenates were sonicated and spun down at 4 °C, 13,000 rpm for 10 min, and supernatant was collected. Protein concentration was determined using DC protein assay. Supernatant was diluted to 2 µg total protein/ml and measured with the serum kit. Serum CCDC3 levels were determined using a sandwich CCDC3 ELISA kit (Mybiosource, San Diego, CA, USA), with 1:2 diluted serum using serum matrix. Cholecystokinin (CCK) levels were determined by a competitive ELISA (Sigma Aldrich, Zwijndrecht, the Netherlands) with 1:8 diluted serum using 1x assay diluent E. Serum insulin was measured as published [24], using the Bio-Plex Pro mouse insulin kit.

### 2.5. RNA Isolation, cDNA Synthesis, and Microarray Hybridization and Analysis

RNA isolation from eWAT, with quality and purity checked and approved, was performed as published [26]. For transcriptome analysis, as part of a larger hybridization experiment, Agilent mouse whole genome microarrays were used (8×60 K, G4852A, Agilent Technologies, Santa Clara, CA, USA). Preparation of the samples, microarray hybridization, and washing was conducted according to the manufacturer’s protocol with a few modifications as described previously [30,31]. Briefly, cDNA was synthesized using 200 ng eWAT RNA with the Agilent low input Quick amp labelling kit without addition of spikes (10 randomly selected mice per group). All samples were individually labelled with Cy5, while for the reference pool, 5 random samples per intervention group were labelled with Cy3 and pooled on an equimolar basis. After hybridization and washing, arrays were covered with ozone-barrier slides and scanned. Signals were quantified using Feature Extraction version 10.7.3 (Agilent), followed by quality control and normalization as published [32]. Microarray data are deposited in Gene Expression Omnibus under accession number GSE53802. In total, 30,733 probes were considered expressed. Differential gene expression was analyzed by unpaired Students’ t-test, and 354 probes were considered significant (*p* < 0.01). We focused on the significant genes with an absolute fold change (ratio of OxR over Control) > 1.25. Interpretation of functional changes was essentially done by classifying genes based on Gene Ontology annotation and pathway analysis using Metacore (Thomson Reuters, New York, NY, USA). Initial categorization was refined using biological databases and scientific literature. As processes overlap, we bundled some processes and renamed them.

### 2.6. Reverse Transcription Quantitative PCR

One µg of total RNA of all individual samples was used for cDNA synthesis using the iScript cDNA synthesis kit (Bio-Rad). Reverse transcription quantitative PCRs (RT-qPCR) were performed as described [22]. Data were normalized using the mean of the reference genes Ribosomal protein S15 (Rps15) and β-2 microglobulin (B2m), which were chosen based on stable and appropriate gene expression levels by microarray analysis, and least CV over all samples. Primers used and their sequences are described in Supplementary Table S2.

### 2.7. Statistical Analysis

**Figure 1:**
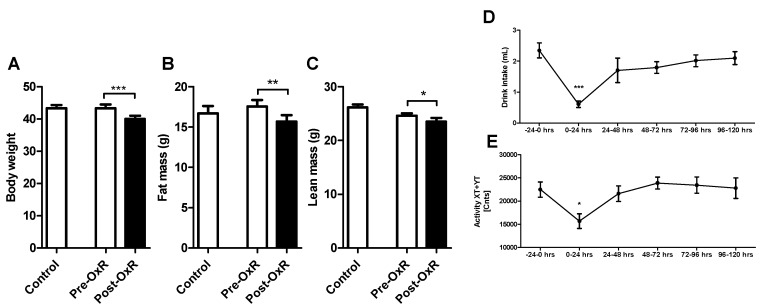
Physiological characteristics of C57BL/6J mice upon oxygen restriction. (**A**) Body weight, (**B**) fat mass, and (**C**) lean mass of control and oxygen restricted (OxR) obese C57BL/6J mice (n = 12/group). Pre-OxR is before intervention with OxR, and Post-OxR indicates after OxR intervention (n = 6/group). (**D**) 24 h drink intake, and (**E**) 24 h activity preceding (–24–0 h) and following 5 days OxR intervention (n = 8–12). Data are shown as mean ± standard error of the mean (SEM); * *p* < 0.05, ** *p* < 0.01, *** *p* < 0.001.

Data are provided as mean ± standard error of the mean (SEM). Significance of difference between Control and OxR was determined by unpaired Students’ *t*-test when data was normally distributed, or alternatively, by Mann–Whitney U-test (WAT lactate and blood glucose levels). For comparison within a group (Figure 1A–C) a paired Students‘ *t*-test was performed. For comparison over time (Figure 1D,E) an One-way ANOVA was performed in combination with Dunnett’s multiple comparison *post hoc* test. We used Graphpad Prism 5.03 (GraphPad Software, San Diego, CA, USA) for analyses and visualization. Differences were considered significant at *p* < 0.05.

## 3. Results

### 3.1. Five Days of Oxygen Restriction (OxR) Altered Physiological Parameters

After 12 weeks of high fat diet feeding, all mice were characterized as obese, with a mean fat mass of 38.9% of body weight (BW). There were no significant differences between control and OxR mice in BW, fat mass, and lean mass (Figure 1A–C) before the start of the intervention. Five days OxR intervention reduced BW significantly, which was accompanied with a significant decline in mean absolute fat and lean mass (Figure 1A–C), while relative fat mass remained similar (~38.5% of BW). Drink intake and activity levels were significantly decreased as a first response to OxR within the first day and returned to being non-significantly different from baseline levels (Figure 1D–E).

**Figure 2:**
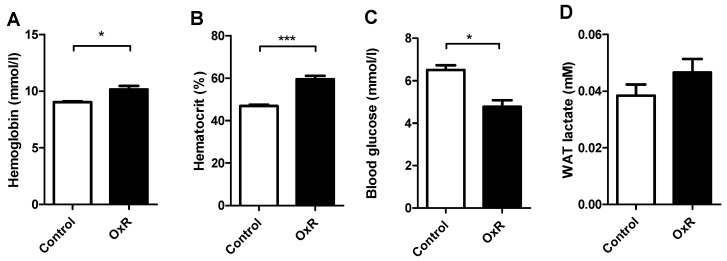
Metabolic effects of oxygen restriction (OxR) intervention. (**A**) OxR increased hematocrit (n =1 2), and (**B**) hemoglobin levels (n = 5–6) in blood, (**C**) decreased blood glucose levels (n = 12), without (**D**) eWAT changes in lactate levels (n = 10). Data are shown as mean ± SEM; * *p* < 0.05, *** *p* < 0.0001.

As an indication for reduced systemic oxygen supply, we measured circulating haemoglobin and haematocrit levels. These were indeed significantly increased as a result of OxR (Figure 2A,B). Furthermore, blood glucose levels were significantly decreased by OxR (Figure 2C), while lactate levels in epididymal WAT remained similar (Figure 2D). This suggests a switch to glycolytic metabolism, indicative for tissue hypoxia and its metabolic adaptations. Serum insulin levels were not affected (Control: 9.61 ± 1.72, OxR: 9.31 ± 1.88 ng/ml; n = 10), but these high levels indicate an insulin resistant state.

### 3.2. Oxygen Restriction (OxR) Did Not Increase Macrophage Infiltration in White Adipose Tissue (WAT), but Decreased WAT Mitochondrial Density

**Figure 3:**
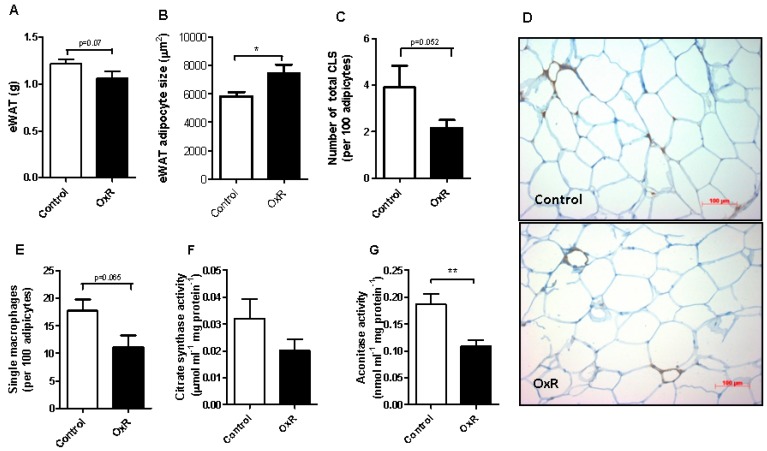
White adipose tissue (WAT) histology and inflammation. Epididymal white adipose tissue (**A**) weight, (**B**) mean adipocyte cell area, (**C**) number of crown-like structures (CLS) assessed by quantification of MAC-2 staining in Control and Oxygen restriction (OxR) mice (n = 12), with (**D**) representative pictures illustrating levels of CLS in Control and OxR-exposed mice. (**E**) number of single macrophages. (**F**) Citrate synthase (n = 6–7) and (**G**) aconitase activity (n = 10) in eWAT. Data are shown as mean ± SEM; * *p* < 0.05, ** *p* < 0.01.

We next focused our attention on epididymal WAT (eWAT). EWAT weight tended to be decreased by OXR (Figure 3A), but adipocyte size was significantly increased (Figure 3B), without altered peroxisome proliferator activated receptor gamma (Pparg) expression (Supplementary Table S3). The number of CLS (Figure 3C,D) as wells as the number of single macrophages (Figure 3E) in eWAT tended to be decreased, but this decrease did not reach statistical significance. This shows that inflammation is not triggered in OxR-exposed mice. The level of citrate synthase, a TCA cycle enzyme, that is used as a marker for mitochondrial density, was not affected by OxR (Figure 3F). Aconitase activity, a TCA cycle enzyme that is sensitive to reactive oxygen species, was significantly decreased in eWAT in response to OxR (Figure 3G).

### 3.3. Gene Expression in Epididymal White Adipose Tissue (eWAT) was Altered Between Oxygen Restriction (OxR) and Control Mice

**Figure 4:**
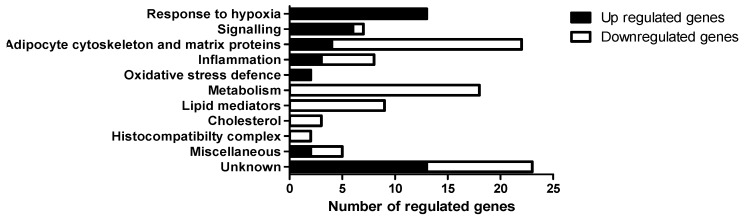
Categorization of the 112 unique genes differentially regulated (*p* < 0.01, absolute fold change >1.25) by oxygen restriction (OxR). Filled black bars represent up regulated genes and open bars represent down regulated genes.

The whole genome eWAT transcriptome after OxR was compared to control mice, which resulted in 69 unique down-regulated genes and 43 unique up-regulated genes with an absolute fold change >1.25 (Supplementary Tables S4 and S5). Up-regulated genes were grouped into five categories, of which response to hypoxia (OxR) and signalling were the top two regulated processes (Figure 4).

**Figure 5:**
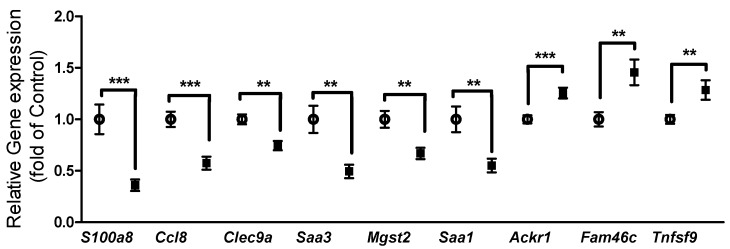
Oxygen restriction (OxR) mostly downregulated white adipose tissue (WAT) inflammatory-related transcripts. Open circles: control, filled squares OxR mice. Data are shown as mean ± SEM (n = 10). ** *p* < 0.01, *** *p* < 0.001.

Down-regulated genes were grouped into six main categories with adipocyte cytoskeleton and membrane proteins, together with metabolism, containing most genes (Figure 4). Furthermore, we identified inflammatory genes among the regulated genes. We identified nine inflammation related transcripts (Figure 5). Six of these transcripts were downregulated, which were in order of significance: the pro-inflammation-related transcripts S100 calcium binding protein A8 (S100a8), Chemokine (C-C motif) ligand 8 (Ccl8 also known as Mcp2), C-type lectin domain family 9 member A (Clec9a), Serum amyloid A3 (Saa3), Microsomal glutathione S-transferase 2 (Mgst2), and Serum amyloid A1 (Saa1). The three upregulated transcripts were Atypical chemokine receptor 1 (Ackr1), family with sequence similarity 46, member C (Fam46c), and tumor necrosis factor (ligand) superfamily, member 9 (Tnfsf9), with, respectively, anti-inflammatory and immune homeostatic properties.

**Figure 6:**
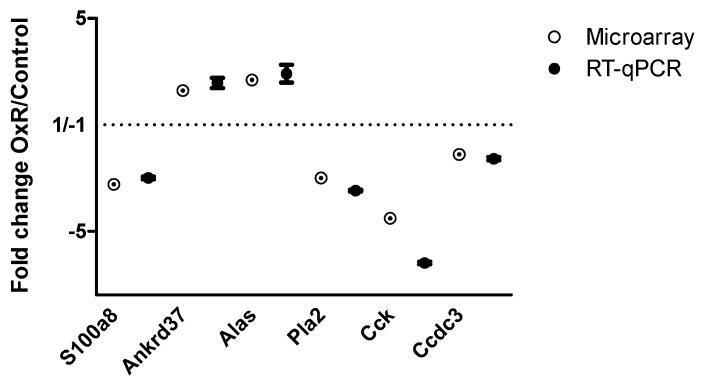
Confirmation of significant microarray results by reverse transcription quantitative PCR. Fold change in gene expression level by oxygen restriction (OxR) over values observed in control group of S100a8, Ankrd37, Alas, Pla2g2e, cholecystokinin (Cck), and coiled coil domain containing 3 (Ccdc3) was analyzed by RT-qPCR (closed circles, n = 10), relative to stable reference genes B2m and Rps15. Data obtained with microarray analysis is shown for comparison by open circles. Data are shown as mean ± SEM.

Genes from four functional categories were selected and their expression was confirmed by RT-qPCR. They represent inflammation (S100a8), adaptation to OxR (ankyrin repeat domain 37 (Ankrd37) and aminolevulinic acid synthase (Alas), lipid mediator (Pla2g2e), as well as the adipokines cholecystokinin (Cck) and coiled-coil domain containing 3 (Ccdc3, also known as Favine) (Figure 6).

### 3.4. Metabolic Serum Adipokines Affected by Oxygen Restriction (OxR)

**Figure 7:**
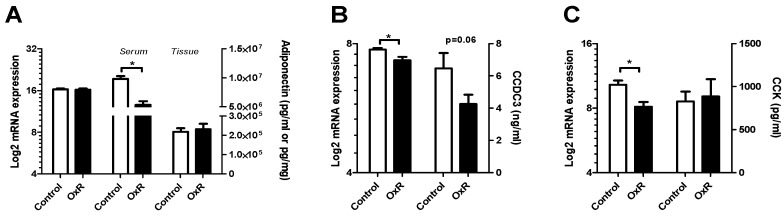
White adipose tissue (WAT) metabolism-associated transcript and protein expression levels and corresponding serum levels. (**A**) Adiponectin levels, (**B**) coiled-coil domain containing 3 (Ccdc3) levels, and (**C**) cholecystokinin (Cck) levels as analysed by transcript levels using gene expression microarrays (left), their serum levels (middle/right), and their tissue level. Open bar: control, closed bar oxygen restriction (OxR) mice. Data are shown as mean±SEM (n = 8–10). * *p* < 0.05.

OxR reduced serum adiponectin levels (Figure 7A), without altering eWAT transcript levels (Figure 5) or tissue protein levels (Figure 7A). Serum CCDC3 levels showed a downward trend by OxR (Figure 7B) corresponding to its decreased transcript levels (Figure 5). Serum CCK levels were not affected by OxR (Figure 7C).

## 4. Discussion

Diet-induced obese C57BL/6J mice, housed at thermoneutrality and additionally challenged by exposure to ambient OxR (13%) for five days, had a systemic decrease of oxygen availability as shown by increased circulating Hb and haematocrit levels, and decreased serum glucose levels. In WAT, lactate levels remained unaltered, while aconitase activity as well as the gene expression profiles in eWAT decreased, where genes indicative of hypoxia are up-regulated, including hypoxia-marker genes Ankrd37 [33] and Alas2 [34] supporting the whole body hypoxic effects. Remarkably, in WAT we found no significant differences in the number of CLS representing tissue inflammation. If anything, a trend towards a decrease rather than an increase was seen. This was supported by a decrease in key marker genes of inflammation, including S100a8, Saa3, and Ccl8. OxR induced alterations in serum levels of adiponectin and CCDC3 as well as WAT tissue levels of CCK and CCDC3. These results suggest that tissue hypoxia affects WAT, but that hypoxia most likely is not the trigger that leads to inflammation of WAT in obese mice, confirming previous results obtained in lean mice [35]. In more detail, to examine whether hypoxia acts as a trigger for WAT dysfunction, male C57BL/6J mice fed a chow diet were housed at 21 °C and exposed to environmental hypoxia (8% O_2_) to challenge WAT. Compared to control mice exposed to an ambient level of 21% O_2_, these mice, surprisingly, showed a reduced, rather than an increased, WAT inflammation [35]. However, translation to an obese condition is difficult, since these mice were lean and not obese, and it is expected that adipocyte sizes were in the normal range due to low fat chow feeding. In contrast, in our study, diet-induced obese mice were housed at thermoneutrality, which increases adipose tissue mass and adipocyte size, but simultaneously excludes thermogenic metabolism as a confounder [24], i.e., mice were unable to compensate by decreasing their metabolic rate upon the hypoxic challenge.

Together, both these experiments, with quite different conditions, seem to indicate that WAT hypoxia is a consequence of impaired adipose tissue function rather than a cause.

Induction of inflammation related transcripts as Tnf-a, interleukin 1 (Il-1), interleukin 6 (Il-6), monocyte chemotactic protein 1 (MCP1), plasminogen activator inhibitor 1 (PAI-1), macrophage migration inhibitory factor (MIF), inducible nitric oxide synthase (iNOS), matrix metallopeptidase 9 (MMP9), and matrix metallopeptidase 2 (MMP2) indicate that hypoxia is able to induce inflammation in primary adipocytes and cell lines in vitro [16,17,18]. However, we observed that none of these genes were regulated in WAT due to OxR intervention in vivo (Supplementary Table S3). We rather observed a down regulation of inflammation associated genes S100a8, Ccl8, Clec9a, Saa3, Mgst2, and Saa1, and consistently observed a tendency for a decrease in CLS or single macrophages in OxR mice, rather than an increase. These findings agree with recent in vitro findings, showing a reduction of the NF-kB signalling and MCP-1 secretion in human primary adipocytes due to hypoxia [36]. Adipocytes are major producers of serum amyloid A (SAA) family members in the non-acute phase in humans [37], especially hypertrophic adipocytes [38] that are known to be associated with obesity and insulin resistance [39]. Moreover, WAT encoded and secreted SAA3 is linked to attraction of monocytes and thus an inflammatory state [40]. This function agrees with the reduced expression levels of various transcripts of Saa and other markers of WAT inflammation in association with an absence of increased number of CLS that was observed here. This is further supported by the upregulation of Ackr1, which encodes a chemokine scavenger receptor that limits chemokine availability and leucocyte recruitment [41]. The effect of the upregulation of Tnfsf9 is difficult to define, because TNFSF9, also known as CD37 ligand and 4-1BBL, has a cell context dependent role in (auto) immune regulation [42], including type I diabetes [43], and in immune system homeostasis [44]. Overall, our data do not support upregulation of inflammation by hypoxia.

Mitochondrial aconitase is a TCA cycle enzyme that converts citrate into isocitrate. It contains a [4Fe4S]^2+^ iron sulphur cluster in its catalytic site. Mitochondrial aconitase is sensitive to inactivation by oxidative stress, because its iron-sulphur cluster as well as specific cysteine residues are readily oxidized by reactive oxygen species (ROS) as well as reactive nitrogen species (RNS) [45,46,47]. Mitochondrial aconitase activity is therefore used as a marker for oxidative stress [48]. Chronic hypoxia has been shown to induce both ROS and RNS [49,50]. This may potentially explain the significantly reduced WAT aconitase activity that was observed here. Reduced aconitase function will result in increased citrate levels. Citrate may be transported into the cytosol, where it can be converted to acetyl-CoA and then Malonyl-CoA for lipid synthesis. As an inhibitor of carnitine palmitoyl transferase 1A (CPT1A), Malonyl-CoA will limit fatty acid import into WAT mitochondria and subsequent fatty acid beta-oxidation. This will reduce oxygen demand. Citrate has also been shown to inhibit pyruvate dehydrogenase [51,52], limiting pyruvate import into mitochondria. This will also result in a decreased WAT oxygen demand.

Hypoadiponectinemia is a biochemical hallmark in the pathogenesis of obesity-related disorders [53,54]. Here, we demonstrated that serum adiponectin is markedly decreased by OxR in vivo. This supports findings in vitro that hypoxia decreases adiponectin expression in adipocytes [55,56]. In contrast to the in vitro observations under more extreme oxygen levels, we observed no change in adiponectin transcript nor protein in eWAT of obese mice exposed to OxR. Circulating adiponectin levels are controlled at the level of release from adipocytes [4], but the major player ERP44 was not differentially expressed by OxR (data not shown). An explanation of our data might be that OxR primarily affects adiponectin clearance. On the other hand, under obese conditions, adiponectin protein and mRNA are low in eWAT [18], which may render eWAT less sensitive to further reduction in cellular adiponectin levels. Other WAT depots may be more sensitive to OxR and may contribute to a larger extend to decreased serum adiponectin levels.

OXR reduced CCK eWAT gene expression, with no effect on serum levels. Expression of CCK in WAT was first shown in visceral WAT of a non-obese subject [57]. CCK is involved in the regulation of appetite. A well-known symptom of OxR is reduction of food intake [58,59]. Reduced Cck transcript levels are therefore remarkable as CCK signals short term satiety. On the other hand, CCK levels may be decreased to counteract hypoxia-induced satiety, possibly in response to a decreasing WAT mass. In that case, because no changes were seen in serum CCK levels, satiety is most likely regulated via nervous signalling as is the case for CCK signalling from the gut [60]. This has not been studied in WAT. Finally, OxR decreased Ccdc3 transcript levels in eWAT, which correlated to decreased CCDC3 serum levels. It is tempting to speculate that this links to its WAT vessel origin [61], with functional implications that need further investigation.

## 5. Conclusions

In summary, five days of reduced oxygen availability did not increase inflammation in eWAT, but rather suggests a decrease in obese mice. Furthermore, a decreased serum level of insulin sensitizing adiponectin was seen. Decreased circulating CCDC3, and adiponectin await confirmation as markers for systemic or WAT hypoxia.

## Supplementary Materials

The following are available online at https://www.mdpi.com/2073-4425/10/5/359/s1, Table S1: Diet composition, Table S2: Primers and annealing temperature for RT-qPCR, Table S3: Marker gene expression for adipogenesis-, angiogenesis-, hypoxia-, and inflammation-related processes, Table S4: Genes downregulated by OxR with an absolute fold change > 1.25 and *p* < 0.01, Table S5: Genes up regulated by OxR with a fold change >1.25 and *p* < 0.01
