# Supplementary material for: White Adipose Tissue Response of Obese Mice to Ambient Oxygen Restriction at Thermoneutrality: Response Markers Identified, but no WAT Inflammation
Source: Genes (Basel). 2019 May 10;10(5):359. doi: 10.3390/genes10050359 (PMC6562665; doi:10.3390/genes10050359)
Supplement: Supplementary file 1 [file genes-10-00359-s001.zip › Supplementary Table S1.pdf]

Supplementary Table S1 Diet composition

Components	LFD	HFD
Casein	220	267
Wheat starch	386.5	172.5
Maltodextrin	100	100
Dextrose	50	50
Sucrose	100	100
Fats*	43	210
Cholesterol	0.03	0.097
Cellulose	50	50
Mineral mixture	35	35
Vitamin mixture	10	10
Choline bitartrate	2.5	2.5
L-cysteine	3	3
Energy (kcal kg^{-1})	3865	4700
Percentage of energy from carbohydrate	66.9	36.8
Percentage of energy from fat	10.0	40.2
Percentage of energy from protein	23.1	23.0

Values are in grams per kilogram, unless stated otherwise. Energy content is adjusted for energy from the vitamin mix. *Fats consist of 18% (w/w) coconut oil, 70% sunflower oil, and 12% flaxseed oil. LFD as published (Hoevenaars, et al., 2012; ref. 26).
