# Supplementary material for: White Adipose Tissue Response of Obese Mice to Ambient Oxygen Restriction at Thermoneutrality: Response Markers Identified, but no WAT Inflammation
Source: Genes (Basel). 2019 May 10;10(5):359. doi: 10.3390/genes10050359 (PMC6562665; doi:10.3390/genes10050359)
Supplement: Supplementary file 1 [file genes-10-00359-s001.zip › Supplementary Table S2.pdf]

Supplementary Table S2 Primers and annealing temperature for RT-qPCR

Gene	Accession	Forward 5'-3'	Reverse 5'-3'	T (°C)
Alas	NM_001102446	TCAGACACAATGACCCAGGC	CGAAGGTCAGGGCTCCATAC	58
Ankrd37	NM_001039562.1	CAGGTGCTGACCTCAACCAAC	CCAAGCAAGATCTTCAGCTGTC	58
Ccdc3	NM_028804.1	CCCGGCTCAACCTTACTGG	GAAATTGACTCCGTGTGGCA	56.5
Cck	NM_031161	ACTGCTAGCGCGATACATCC	CATCCAGCCCATGTAGTCCC	58
S100a8	NM_013650	ACTTCGAGGAGTTCCTTGCG	TGCTACTCCTTGTGGCTGTC	58
Il1β	NM_008361	TGCACTACAGGCTCCGAGAT	AGGCCACAGGTATTTTGTCGT	58
Pla2	NM_012044	GGACGAGACGGATTGGTGTT	AGTGTTCAGGTTGTGGCGAA	58
Slc2a1/ Glut1	NM_011400.3	TATCGTCAACACGGCCTTCA	TGGCCACGATGCTCAGATAG	58
*B2m**	NM_009735	CCCCACTGAGACTGATACATACGC	AGAAACTGGATTTGTAATTAAGCAGGTTC	60
*Rps15**	NM_009091	CGGAGATGGTGGGTAGCATGG	ACGGGTTTGTAGGTGATGGAGAAC	58

*genes denoted with an asterisk were together used as reference genes for RT-qPCR. Annealing temperature for RT-qPCR is provided in degrees Celsius.
