# Supplementary material for: White Adipose Tissue Response of Obese Mice to Ambient Oxygen Restriction at Thermoneutrality: Response Markers Identified, but no WAT Inflammation
Source: Genes (Basel). 2019 May 10;10(5):359. doi: 10.3390/genes10050359 (PMC6562665; doi:10.3390/genes10050359)
Supplement: Supplementary file 1 [file genes-10-00359-s001.zip › Supplementary Table S3.pdf]

Supplementary Table S3. Marker gene expression in eWAT for adipogenesis-, angiogenesis-, hypoxia-, and inflammation-related processes between OxR and Control mice.

Gene symbol	Gene name/marker	FC	*P*-value
Adipogenesis			
Pparg	peroxisome proliferator activated receptor gamma	1.1	0.32
Slc2a4	solute carrier family 2 (facilitated glucose transporter), member 4; a.k.a. Glucose transporter 4 (*Glut4*)	1.2	0.55
Zfp423	zinc finger protein 423	-1.1	0.62
Angiogenesis			
Angpt1	angiopoietin 1	-1.1	0.34
Angpt2	angiopoietin 2	1.3	0.10
Epcam	epithelial cell adhesion molecule	-1.2	0.49
Flt1	FMS-like tyrosine kinase 1	1.1	0.22
Kdr	kinase insert domain protein receptor	1.1	0.36
Tie1	tyrosine kinase with immunoglobulin-like and EGF-like domains 1	1.0	0.69
Vcam1	vascular cell adhesion molecule 1	-1.1	0.32
Vegfa	vascular endothelial growth factor A	1.2	0.24
Vegfb	vascular endothelial growth factor B	1.1	0.38
Hypoxia			
Slc2a1	solute carrier family 2 (facilitated glucose transporter), member 1; a.k.a. Glucose transporter 1 (*Glut1*)	1.2	0.40
Spp1	Secreted phosphoprotein 1; a.k.a. Osteopontin (*Opn*)	-1.1	0.74
Inflammation			
Adgre1	adhesion G protein-coupled receptor E1; a.k.a. F4/80	n.d.	
Arg1	arginase, liver; M2 macrophage marker	1.2	0.37
Ccl2	chemokine (C-C motif) ligand 2	1.2	0.21
Cybb	cytochrome b-245, beta polypeptide; a.k.a. *Nox2*	1.0	0.73
Il1	interleukin 1 complex	n.d.	
Il6	interleukin 6	1.4	0.06
Itgam	integrin alpha M; a.k.a. CD11b	1.2	0.22
Itgax	integrin alpha X; a.k.a. CD11c	1.0	0.93
Mif	macrophage migration inhibitory factor (glycosylation-inhibiting factor)	1.1	0.26
Mmp2	matrix metallopeptidase 2	1.0	0.91
Mmp9	matrix metallopeptidase 9	1.1	0.39
Nos2	nitric oxide synthase 2, inducible; M1 macrophage marker	1.1	0.41
Tnf	tumor necrosis factor	1.1	0.52
Serpine1	serine (or cysteine) peptidase inhibitor, clade E, member 1; a.k.a. PAI-1	1.1	0.73

Categorized transcripts per process were subsequently sorted based on alphabetical order. FC: fold change of oxygen restricted (OxR) group over control group; a.k.a.: also known as; n.d.: no detectable expression levels. In case multiple probes are present on the Agilent gene expression array, the one with highest FC with lowest p-value is shown.
