# Supplementary material for: White Adipose Tissue Response of Obese Mice to Ambient Oxygen Restriction at Thermoneutrality: Response Markers Identified, but no WAT Inflammation
Source: Genes (Basel). 2019 May 10;10(5):359. doi: 10.3390/genes10050359 (PMC6562665; doi:10.3390/genes10050359)
Supplement: Supplementary file 1 [file genes-10-00359-s001.zip › Supplementary Table S4.pdf]

Supplementary Table S4. Genes down regulated by OxR with a fold change <-1.25 and p< 0.01

Gene symbol	Gene name	FC	P-value
Adipocyte cytoskeleton and matrix proteins			
1100001G20Rik	RIKEN cDNA 1100001G20 gene	-2.1	0.0073
A2ld1	Gamma-glutamylamine cyclotransferase	-1.3	0.0033
Acta1	Actin, alpha 1, skeletal muscle	-1.7	0.0022
Col4a5	Collagen, type IV, alpha 5	-1.3	0.0070
Gkn3	Gastrokine 3	-1.5	0.0003
Hapln3	Hyaluronan and proteoglycan link protein 3	-1.3	0.0058
Itgbl1	Integrin, beta-like 1	-1.4	0.0030
Krtap11-1	Keratin associated protein 1-1	-1.4	0.0013
Lctl	Lactase-like	-1.6	0.0019
Lrtm1	Leucine richrepeats and transmembrane domains 1	-1.6	0.0004
Maoa	Monoamine oxidase A	-1.3	0.0008
Mme	Membrane metallo endopeptidase	-1.5	0.0009
Myom1	Myomesin 1	-1.3	0.0057
Serpina3b	Serine (or cysteine) peptidase inhibitor, clade A, member 3B	-1.4	0.0071
Serpina3m	Serine (or cysteine) peptidase inhibitor, clade A, member 3M	-1.7	0.0004
Tinag	Tubulointerstitial nephritis antigen	-1.8	0.0013
Tpm1	Tropomyosin 1, alpha	-1.4	0.0026
Trdn	Triadin	-1.9	0.0082
Metabolism			
Acoxl	Acyl-Coenzyme A oxidase-like	-1.4	0.0061
Aldh1a7	Aldehyde dehydrogenase family1, subfamily A7	-1.7	0.0095
Apoc4	Apolipoprotein C-IV	-1.4	0.0005
Atp1a4	ATPase, Na+/K+ transporting, alpha 4 polypeptide	-1.7	0.0087
Bcat1	Branched chain aminotransferase 1, cytosolic	-1.3	0.0066
Cox8b	Cytochrome c oxidase subunit VIIIb	-1.9	0.0055
Fabp12	Fatty acid binding protein 12	-1.4	0.0042
Glul	Glutamate-ammonia ligase (glutamine synthetase)	-1.3	0.0026
Grb14	Growth factor receptor bound protein 14	-1.6	0.0003
Gys2	Glycogen synthase 2	-1.8	0.0029
Lrpap1	Low density lipoprotein receptor –related protein associated protein 1	-1.3	0.0031
Mpst	Mercaptopyruvate sulfurtransferase	-1.5	0.0020
Nudt14	Nudix (nucleoside diphosphate linked moiety X)-type motif 14	-1.3	0.0001
Ppic	Peptidylprolyl isomerase C	-1.3	0.0028
Rwdd3	RWD-domain containing 3	-1.4	0.0009
Sfxn5	Sideroflexin 5	-1.3	0.0011
Tph2	Tryptophan hydroxylase 2	-2.7	0.0033
Tst	Thiosulfate sulfurtransferase, mitochondrial	-1.4	0.0065
Lipid mediators			
Cyp2c44	Cytochrome P450, family 2, subfamily c polypeptide 44	-1.8	0.0003
D3Bwg0562e	DNA segment, Chr3, Brigham & Women's Genetics 0562 expressed	-1.9	0.0096
Enpp5	Ectonucleotide pyrophosphatase/phosphodiesterase 5	-1.4	0.0099
Hpgd	Hydroxyprostaglandin dehydrogenase 15 (NAD)	-1.3	0.0004
Mgst2	microsomal glutathione S-transferase 2	-1.5	0.0033

Gene symbol	Gene name	FC	P-value
Pemt	Phosphatidylethanolamine N-methyltransferase	-1.3	0.0075
Pla2g2e	Phospholipase A2, group IIE	-2.5	0.0001
Pla2g5	Phospholipase A2, group V	-1.9	0.0056
Slco3a1	Solute carrier organic anion transport family, member 3a1	-1.5	0.0003
Inflammation			
Ccl8	Chemokine (C-C motif) ligand 8	-1.8	0.0002
Clec9a	C-type lectin domain family 9, member a	-1.4	0.0014
S100a8	S100 calcium binding protein A8 (calgranulin A)	-2.8	0.0001
Saa1	Serum amyloid A 1	-1.8	0.0077
Saa3	Serum amyloid A 3	-2	0.0025
Cholesterol			
Dhcr24	24-dehydrocholesterol reductase	-1.6	0.0006
Nsdhl	NAD(P) dependent steroid dehydrogenase-like	-1.3	0.0078
Tm7sf2	Transmembrane 7 superfamily member 2	-1.3	0.0001
Histone regulation/modification			
H2-K1	Histocompatibility 2, K1, K region	-1.3	0.0037
H2-Q10	Histocompatibility 2, Q region locus 10	-1.3	0.0060
Zmynd15	Zinc finger, MYND-type containing 15	-1.4	0.0002
Miscellaneous			
Ccdc3	Coiled coil domain containing 3	-1.4	0.0094
Cck	Cholecystokinin	-4.4	0.0041
Hfe	Hemochromatosis	-1.4	0.0034
Unknown			
6030419C18Rik			
9130204L05Rik		-1.8	0.0047
A_55_P1981992		-1.3	0.0018
B230311B06Rik		-1.3	0.0032
BC054084		-1.4	0.0023
Chr1:138579972-138587381_F		-1.5	0.0026
Chr10:93819821-93976621_F		-1.3	0.0008
chr8:89996710-90049453_F		-1.3	0.0050
ENSMUST00000090166		-1.4	0.0007
NAP060515-1		-1.3	0.0019

Processes are shown in the order as presented in Figure 3. Categorized transcripts per process were subsequently sorted based on alphabetical order.
