# Supplementary material for: White Adipose Tissue Response of Obese Mice to Ambient Oxygen Restriction at Thermoneutrality: Response Markers Identified, but no WAT Inflammation
Source: Genes (Basel). 2019 May 10;10(5):359. doi: 10.3390/genes10050359 (PMC6562665; doi:10.3390/genes10050359)
Supplement: Supplementary file 1 [file genes-10-00359-s001.zip › Supplementary Table S5.pdf]

Supplementary Table S5. Genes up regulated by OxR with a fold change > 1.25 and p<0.01

Gene symbol	Gene name	FC	P value
**Response to OxR**			
Alas2	Aminolevulinic acid synthase 2, erythroid	2.1	0.0013
Ankrd37	Ankyrin repeat domain 37	1.6	0.0008
Beta-s	Hemoglobin subunit beta-1-like	2.1	0.0009
Chrdl1	Chordin-like 1	1.9	0.0017
Cish	Cytokine inducible SH2-containing protein	1.7	0.0094
Edn1	Endothelin 1	1.3	0.0067
Gm5226		2	0.0007
Hba-a1	Hemoglobin alpha, adult chain 1	1.9	0.0045
Hba-a2	Hemoglobin alpha, adult chain 2	1.9	0.0073
Hbb-b1	Hemoglobin, beta adult major chain	2	0.0017
Hbb-b2	Hemoglobin, beta adult minor chain	1.9	0.0005
Slc25a37	Solute carrier family 25, member 37	1.3	0.0003
Slc4a1	Solute carrier family 4 (anion exchanger), member 1	1.5	0.0006
**Signalling**			
1300014I06Rik	PX domain containing 1	1.4	0.0020
Enc1	Ectodermal-neural cortex 1	1.3	0.0087
Hic1	Hypermethylated in cancer 1	1.3	0.0042
Htra3	HtrA serine peptidase 3	1.4	0.0076
Rgs16	Regulation of G-protein sugnalling 16	1.3	0.0082
Tmem198	Transmembrane protein 198	1.3	0.0021
**Adipocyte cytoskeleton and matrix proteins**			
Has2	Hyaluronan synthase 2	1.3	0.0095
St6galnac5	ST6 (alpha-N-acetyl-neuraminyl-2,3-beta-galactosyl	1.5	0.0044
Zdhhc22	Zinc finger, DHCC-type containing 22	2.1	0.0013
Tubb3	Tubulin, beta 3 class III	1.4	0.0049
**Inflammation**			
Darc	Duffy blood group, chemokine receptor	1.3	0.0008
Fam46c	Family with sequence similarity 46, member C	1.4	0.0070
Tnfsf9	Tumor necrosis factor (ligand) superfamily, member C	1.3	0.0091
**Oxidative stress defence**			
Gstm1	Gluthathione S-transferase, mu 1	1.5	0.0023
Gstm4	Gluthathione S-transferase, mu 4	1.4	0.0024
**Miscellaneous**			
Gnat2	Guanine nucleotide binding protein, alpha transducing 2	1.3	0.0058
Prpf4b	PRP4 pre-mRNA processing factor 4 homolog B (yeast)	1.3	0.0026
**Unknown**			
2700081O15Rik		1.3	0.0084
4930415C11Rik		1.4	0.0013
chr1:107866276-107887102 R		1.3	0.0059
chr8:106037800-106070025 F		1.3	0.0044
chr8:74431707-74442440 F		1.3	0.0008
chr10:69819062-69871640 F		1.3	0.0052
chr14:28218945-28241903 R		1.3	0.0019
chr17:47006033-47023558 R		1.3	0.0020
Ensmust00000073543		1.3	0.0066
Fam171b		1.3	0.0076
Gm10447		1.3	0.0021
Gm16516		1.3	0.0005
Gm9992		1.7	0.0001

Processes are shown in the order as presented in Figure 3. Categorized transcripts per process were subsequently sorted based on alphabetical order.
